# Effect of Boron on Hot Ductility and Room-Temperature Tensile Properties of Microalloyed Steels with Titanium and Niobium

**DOI:** 10.3390/ma12142290

**Published:** 2019-07-17

**Authors:** Qi Li, Weijie Liu

**Affiliations:** School of Material Science and Engineering, Northeastern University, Shenyang 110819, China

**Keywords:** hot ductility, tensile property, boron, transverse crack, precipitation

## Abstract

Effect of boron on the hot ductility and room-temperature tensile properties of Ti-Nb-microalloyed steels containing 0.071 wt.% carbon was studied. The thermal stress and thermal strain of continuous casting billets during cooling were simulated via hot tensile tests at the deformation rate of (6 mm/11,000)/s, and the hot ductility of different microalloyed steels was evaluated according to the area reduction of hot tensile specimens. It was found that boron addition was beneficial to improve the hot ductility of continuous casting billets during straightening, and the reduction of area exceeded 60%. The addition of boron, as well as the removal of molybdenum and vanadium, can effectively lower the austenite-to-ferrite transformation temperature and restrain the formation of intergranular ferrite, so as to avoid the brittle zone. Moreover, the room-temperature tensile properties of the steels were explored at different cooling rates after the rolling process. The results showed that as the cooling rate increased from 0.0094 to 0.13 °C/s, the amount of carbonitride precipitate gradually decreased, such as titanium carbide, leading to the relatively low tensile strength. On the other hand, the addition of boron, as well as the removal of Mo and V, promoted the formation of bainite and acicular ferrite, playing an important role in structure strengthening, and compensated for the decrease of tensile strength caused by the low precipitation strengthening.

## 1. Introduction

The hot ductility loss of microalloyed steels during continuous casting is a severe problem and is largely related to the numerous surface cracks formed on the casting billets. At the straightening stage of continuous casting billets, hot ductility is a valid parameter for evaluating the crack sensitivity of steel billets. The hot ductility of continuous casting billets is affected by many factors, including the austenite/ferrite transformation, precipitate phase, and dynamic recrystallization [1,2,3,4]. Furthermore, the variation of hot ductility caused by these microstructure changes is critically associated with the microalloy elements of steels. The variation of microelements also directly affects the room-temperature mechanical properties of steels.

The surface transverse cracks of casting billets were solved at early times by studying the hot ductility behaviors via high-temperature mechanical tests. Suzuki et al. proposed and improved the mechanism of three brittle temperature ranges [5,6,7] in order to solve the cracking of casting billets. When the temperature is between 1200 and 1420 °C (the melting point), the embrittlement is caused by the melting of grain boundaries. As the temperature is within 900–1200 °C, the embrittlement is attributed to the reduction of grain boundary strength due to the sulfides and oxides precipitated at grain boundaries. When the temperature is between 600 and 900 °C, the embrittlement is mainly induced by the intergranular ferrite separated along the original austenite grain boundaries.

The relationship between the third brittle zone and transverse cracks has been explored in detail [8,9,10,11]. The ductility trough was formed below 775 °C because the strain-induced pro-eutectoid ferrite precipitated along the austenite grain boundary, and since the strength of ferrite was weaker than austenite, the stress was concentrated on the softer α phase, which resulted in the cracking along the austenite grain boundary. Above 775 °C, the ductility trough was induced by the grain boundary slip. At the high-temperature side of the ductility trough, the occurrence of dynamic recrystallization led to the ductility recovery, but the precipitation of microalloy carbonitride inhibited the dynamic recrystallization, thereby broadening the ductility trough of steels. At the low-temperature side of the ductility trough, the ductility recovery depended on the formation of abundant ferrite at grain boundaries and inside grains. During the straightening of casting billets, when the ferrite volume fraction at the two phases of austenite and ferrite was above 45%, the hot ductility of casting billets can be effectively improved, and the formation of transverse cracks can be decreased.

The addition of trace elements critically affected the hot ductility of steels. The boron can significantly enhance the hot ductility of steels, mainly because the segregation of boron (B) atoms at the austenite grain boundary delayed the austenite/ferrite transformation, which may avoid the formation of ferrite films at austenite grain boundary and can strengthen the resistance against grain boundary slip during the straightening, improving the hot ductility [12,13]. Fujiwara [14] believed the addition of B can significantly enhance the high-temperature creep strength of steels. Laha [15] uncovered the underlying mechanism and thought the B segregated at grain boundary could enter into the precipitates and change the properties of grain boundary/precipitate interface or matrix/precipitate, thereby inhibiting the microcavity formation and decreasing the tendency of microcracking. Cheng-bin Shi et al. [16] studied the effects of B content on the hot ductility of steels and found the solid solution state of B can replace the coarse boron nitride (BN), thereby improving the hot ductility of steels. Moreover, as the B concentration rose, the ductility trough of the steels gradually disappeared.

The presence of titanium (Ti) alone did not largely affect the crack formation in steels, but the addition of Ti into the steels containing Al, Nb, and V can improve the formation of transverse cracks at corners [17]. The precipitation temperature of TiN was higher and usually initiated at above 1350 °C. Due to the strong binding ability of Ti with C and N, the abundant newly-formed TiN and Ti(C, N) decreased the concentration of free N and thereby reduced the amount of aluminium nitride (AlN) separated at austenite grain boundary, which thereby ameliorated the steel ductility [3,18]. Ti can improve the hot ductility of Nb-containing steels only when the cooling rate is slow enough, but the improvement was not significant when the cooling rate was at 100 K/min [19]. Mintz [17] found that the addition of Ti, especially excessive Ti content (0.12%), into containing V or Nb steels may damage the hot ductility to some extent, which may be caused by the TiC precipitated in the γ phase.

The addition of vanadium (V) was critical in improving the hot ductility of twinning induced plasticity (TWIP) steel, which may be the ability to form tiny VC particles and thereby enhanced the strengthening effect and promoted dynamic recrystallization [20]. However, Salas-Reyes found the loss of hot ductility in high-strength steels at low temperature was related to the coupling between precipitates and gaps, especially V(C, N), which was critical in the formation mechanism of cracks [21]. The addition of molybdenum (Mo) also enhanced the solid solution strengthening and inhibited the dynamic recrystallization, which may be because the solute drag effect decreased the grain boundary mobility and changed the precipitation behaviors of Nb(C, N) [22,23].

The hot ductility of microalloyed steel continuous casting billets has been explored, especially the effects of trace elements. However, the research about improving the hot ductility of steels by changing trace alloy elements has rarely involved the changes of mechanical properties of steels at room temperature. Thus, in this study, the hot ductility and room-temperature mechanical properties of steels were studied.

## 2. Experimental Procedure

The chemical compositions of the four tested steels were listed in Table 1, including steels S1 and S2 cut from continuous casting billets on-site, and steels S3 and S4 with composition optimization through melting in the laboratory. The dosages of four alloy elements (Ti, Mo, V, B) were optimized: the dosage of Ti decreased from 0.112% to 0.085%, Mo and V were removed, and 0.002% B was added.

The tested steel cast ingots were cut into square billets and heated again to 1250 °C and preserved for 2 h before homogenization. The billets were rolled via 6-passes on a Φ450 mm laboratory rolling mill into 12-mm-thick blanks at the initial and final rolling temperatures of 1150 and 900 °C, respectively, and then were air-cooled to room temperature. The blanks were made into specimens for thermal dilatometry specimens and high-temperature tensile specimens. The length direction of the specimens was consistent with the rolling direction of the blanks.

The on-site cooling conditions of the casting billets were approximately simulated via the thermal dilatometry, and the beginning and ending temperatures of γ→α transformation under continuous cooling conditions were measured. The specimens were heated on a thermal expansion instrument (DIL805A/D) at a rate of 10 °C/s to 1200 °C and preserved for 3 min. Then, the specimens were cooled at a rate of 1 °C/s to 910 °C and preserved for 60 s. Finally, the specimens were cooled at a rate of 0.01 °C/s to 600 °C.

**Figure 1:**
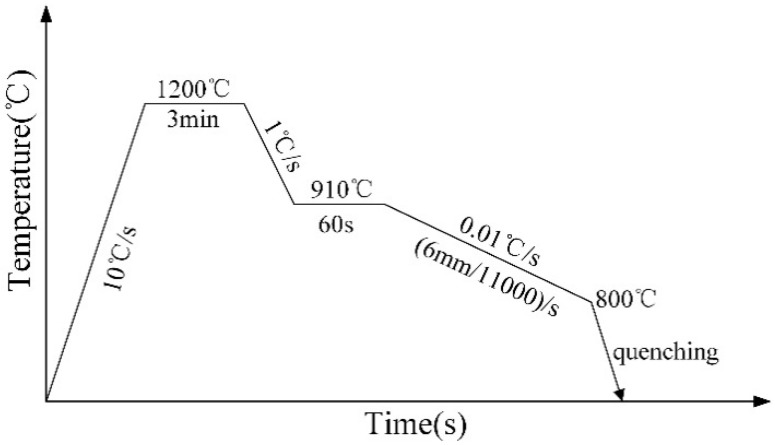
Schematic diagram of thermomechanical test profile for hot tensile test.

The thermo-mechanical field states of the casting billets during cooling were approximately simulated on MMS300 thermal simulation machine so as to study the hot ductility of continuous casting billets. The thermomechanical test profile of hot tensile test is schematically illustrated in Figure 1. The specimens were heated at a rate of 10 °C/s to 1200 °C and preserved for 3 min, and then cooled at a rate of 1 °C/s to the deformation temperature 910 °C. Before deformation, the specimens were held at 910 °C for 60 s to homogenize the temperature. From 910 °C on, the specimens were cooled at a rate of 0.01 °C/s and the deformation rate of (6 mm/11,000)/s with gauge length of 10 mm for continuous deformation, so as to simulate the thermal stress and thermal strain in the casting billets during the cooling process. When fracture initiated, the specimens were rapidly cooled by air jet to room temperature so as to reserve the microstructure upon fracturing.

To clarify the relatively low strength of the head and end of the hot rolled coils at room temperature, the hot rolling process of steel blanks was approximately simulated by using a φ450 mm laboratory rolling mill. The steel billets were heated to 1200 °C, and the starting and ending rolling temperatures were 1150 and 900 °C respectively, with a 3-pass rolling from 12 to 4 mm. After the rolling, the rolled blanks were air-cooled to 600 °C and immediately transferred to an annealing furnace preset to 600 °C (close-door furnace cooling and open-door furnace cooling) or were directly buried in heat-preservation asbestos, so as to simulate the cooling rates at different parts of the hot rolled coils. After cooling to 200 °C, the steel plates were taken out and air-cooled to room temperature. In order to monitor the real-time temperatures of the rolled blanks, WRNK-191 armored thermocouples were inserted the holes drilled at the center of the lateral face of each rolled plate, and the temperature change was monitored in real time using a KM-320 temperature recorder. The materials near the thermocouples were cut off and made into tensile samples, which were tested on a CMT 5105 universal tensile testing machine at the deformation rate of 3 mm/s.

The metallographic specimens were sampled from the high-temperature tensile samples and the hot rolled samples. The samples were corroded with 4% nitric acid alcohol for 15 s and then the microstructures were observed with an MEF4A metallographic microscope. The microstructures and chemical compositions of the precipitate phase were characterized by FEI Tecnai G2F20S–TWIN transmission electron microscope.

## 3. Results

### 3.1. Hot Ductility

The γ→α transformation point of the four tested steels was measured using a thermal expansion instrument (Table 2). Clearly, compared with S1 and S2, the initial temperature of austenite-to-ferrite transformation is significantly lower in S3 and S4 steels, and the ending temperature obviously declines. This is because the addition of B into S3 and S4 steels promotes the dynamic recrystallization of austenite, and the non-equilibrium segregation of B atoms at the austenite grain boundary can delay the austenite-to-ferrite transformation, thereby decreasing the ferrite transition temperature [12,24].

The hot tensile tests of the four tested steels were conducted on an MMS300 thermal simulation machine, shown in Table 3. Due to the limitation in the number of data acquisition points of the thermal simulation machine, after temperature cooling to 800 °C, the S4 steel did not fracture during the tensile process. The S3 and S4 steels with composition optimization show significantly higher elongation (up to 60%) than that of S1 and S2. Moreover, the reduction of area (RA) does not exceed 40% in either S1 or S2, while the reduction of area is above 60% in both S3 and S4. Thus, S3 and S4 both show significantly better reduction of area than S1 and S2, which indicates very good hot ductility.

As reported in [17], during hot tensile tests, when the reduction of area after fracturing is less than 40%, this steel can be considered as low hot ductility. Thus, studying on the hot ductility of steels, the reduction of area usually is selected as the basis and standard to judge the hot brittleness zones. Thus, it can be seen that S3 and S4 steels show high hot ductility and low crack sensitivity. It is noticeable that S4 did not fracture at the end of tensile failure tests (cooling to 800 °C), which indicated better hot ductility.

Moreover, it can be judged on basis of reduction of area and fracturing temperature that, upon the fracturing, S1 and S2 were located at the third brittle zone and showed low hot ductility, indicating the continuous casting billets during straightening and rolling were very prone to cracks and even fractures. On the contrary, S3 and S4 steels showed high hot ductility in this temperature range and kept away from the third brittle zone, thus avoiding the crack formation and expansion.

**Figure 2:**
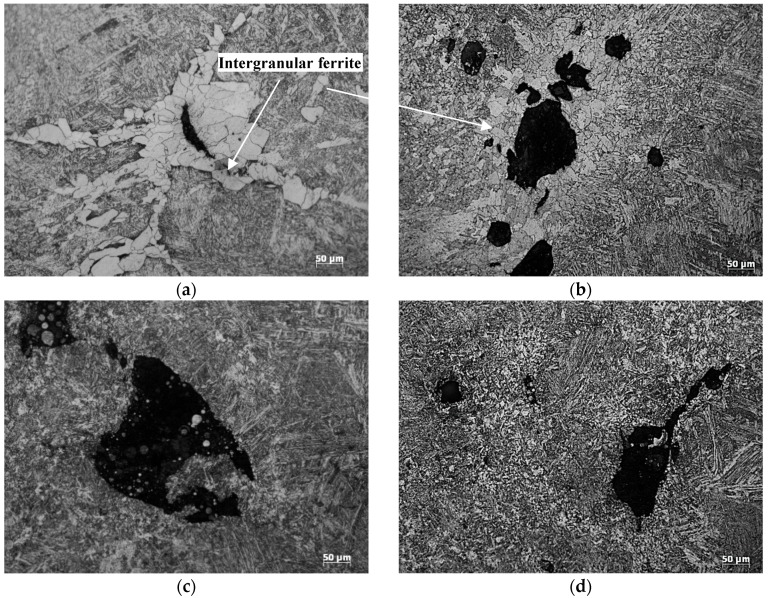
Microstructure near the fractures of hot tensile specimens: (**a**) S1; (**b**) S2; (**c**) S3; (**d**) S4.

To further study the hot ductility behaviors of the steels, the metallography at the fractures of the hot tensile samples are observed by an optical microscope, shown in Figure 2. The sectional metallography of S1 and S2 show the intergranular ferrite structures precipitated along the austenite grain boundary around the cracks and holes. However, no evident ferrite is found around the cavities and microcracks of the metallography of either S3 nor S4, and the microcracks and cavities form along the austenite grain boundary during the hot tensile process. Noticeably, though S4 did not fracture during the hot tensile test, cavities or cracks appeared at the center of sample.

### 3.2. Room-Temperature Microstructures and Mechanical Properties

**Figure 3:**
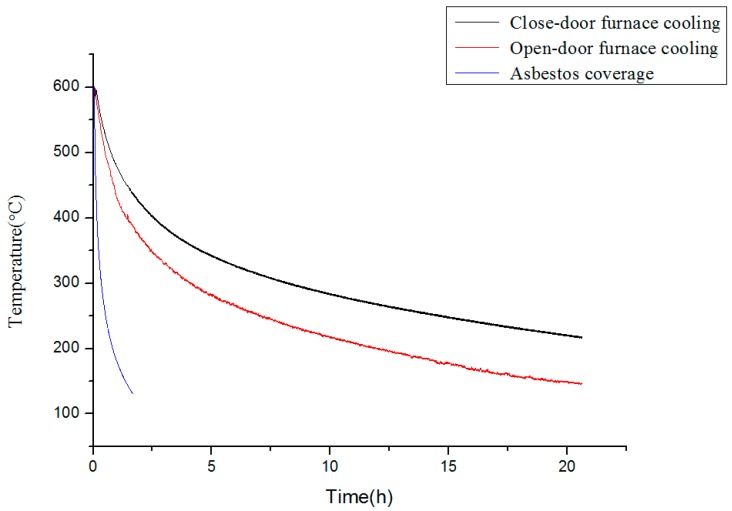
Time–temperature curves of different cooling ways.

To solve the relatively low strength at the head and end of the hot rolled coils, the cooling rates at different parts of the hot rolled coils were simulated. Three ways of cooling were adopted, including asbestos preservation cooling, close-door furnace cooling, and open-door furnace cooling. The temperature–time curves are given in Figure 3. The cooling rate is the highest with the asbestos heat preservation and is 0.13 °C/s on average. The cooling rates of the open-door and close-door furnace cooling ways are 0.0094 and 0.0038 °C/s, respectively.

**Figure 4:**
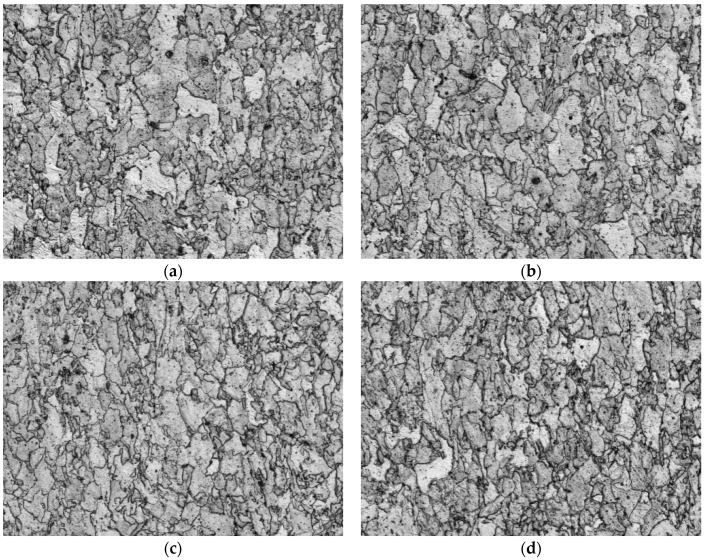
Metallurgical structures under close-door furnace cooling, 500x: (**a**) S1; (**b**) S2; (**c**) S3; (**d**) S4.

**Figure 5:**
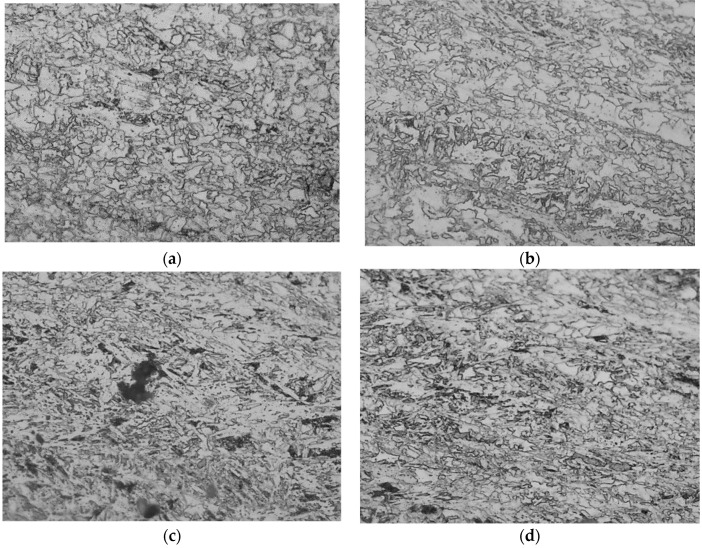
Metallurgical structures under open-door furnace cooling, 500x: (**a**) S1; (**b**) S2; (**c**) S3; (**d**) S4.

**Figure 6:**
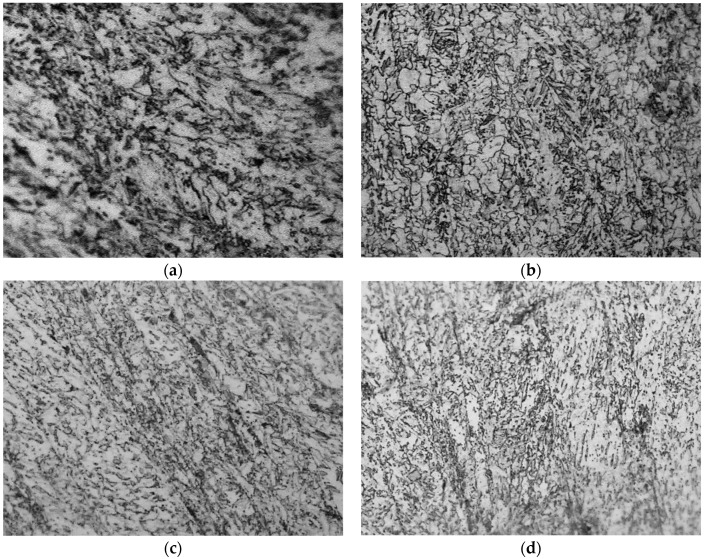
Metallurgical structures under asbestos coverage, 500x: (**a**) S1; (**b**) S2; (**c**) S3; (**d**) S4.

Figure 4, Figure 5 and Figure 6 show the metallographic structures of the four tested steels by the three cooling ways respectively, and Table 4 lists the bainite volume fractions analyzed on Image-pro plus. Clearly, under the close-door furnace cooling, the metallographic characteristics of the four types steels are very similar, which are all equiaxed ferrite + minor acicular ferrite (Figure 4). As the cooling rate increases, the changes of microstructures are not obvious in S1 or S2 steels. However, the amount of acicular ferrite obviously increases in both S3 and S4 experimental steels, and a small amount of bainite is present with a volume fraction of more than 16% (Figure 5). As the cooling rate further increases, the microstructures in S3 and S4 are both fully transformed to bainite and acicular ferrite as well as minor martensitic structures, but S1 and S2 are still dominated by ferrite, and the grain sizes are finer (Figure 6).

The above observations suggest that as the cooling rate increases, the microstructures gradually transform from equiaxed ferrite to acicular ferrite or bainite, and the grain size is reduced accordingly. The bainite and acicular ferrite both have good strength, so the microstructures show the S3 and S4 had excellent mechanical properties at a very fast cooling rate.

**Figure 7:**
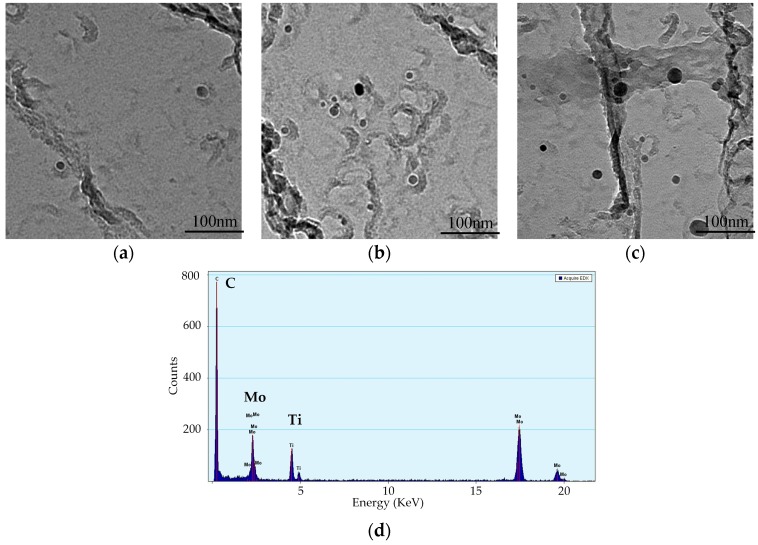
Precipitation distribution of S4 steel at different cooling rates: (**a**) 0.13 °C/s; (**b**) 0.0094 °C/s; (**c**) 0.0038 °C/s; (**d**) energy dispersive spectrometer (EDS) of TiC through carbon extraction replicas with Mo mesh.

**Figure 8:**
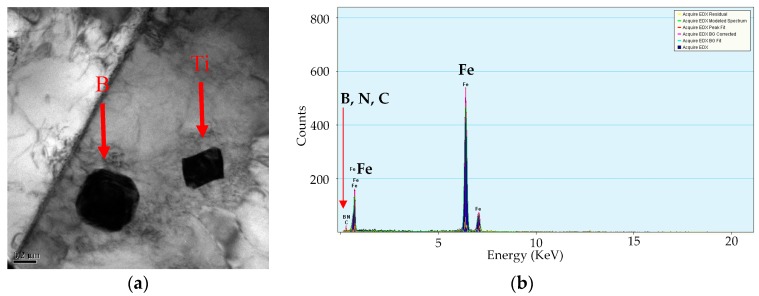
TEM image of S4 steel at the cooling rate 0.0038 °C/s. (**a**) carbide in ferrite grain; (**b**) energy dispersive spectrometer (EDS) of BN.

Figure 7 shows the precipitated phase of S4 steel at different cooling rates observed by TEM. Clearly, the precipitated phase TiC particles are uniformly distributed at the grain boundary and the grains interior of ferrite and are in the size range of 5–30 nm. The precipitation is minor at the cooling rate of 0.13 °C/s (Figure 7a). As the cooling rate gradually decreases, the amount of the precipitate phase TiC increases significantly (Figure 7b,c) and the precipitation size enlarges. Figure 7d shows the EDX images of the precipitated phase TiC. Additionally, S3, the precipitated phase distribution of S3 steel, is similar to that of S4, showing a decrease in the precipitation phase as the cooling rate increases. Moreover, the BN grains are distributed near the ferrite grain boundary (Figure 8a), and EDX images are shown in Figure 8b. The typical microstructure of precipitation, TiN, is also found in Figure 8b.

**Figure 9:**
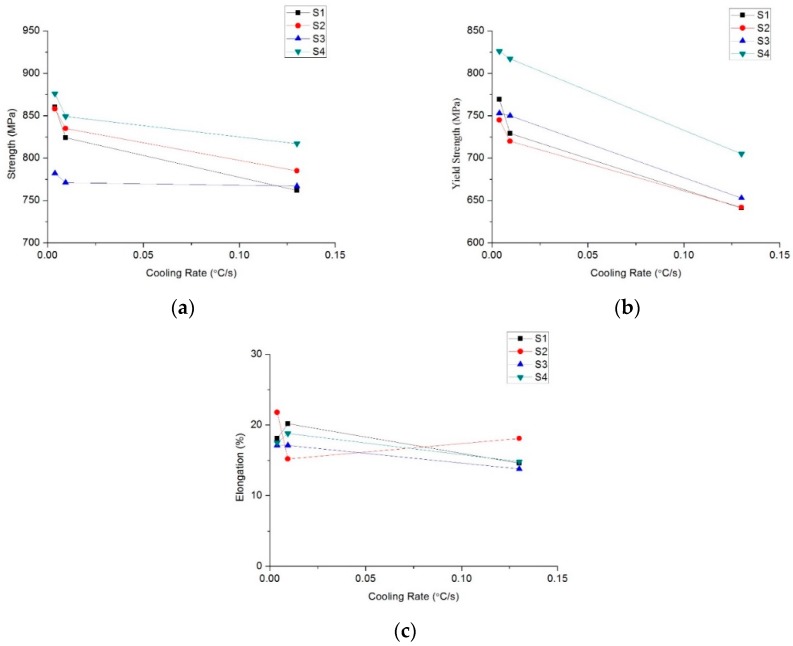
Mechanical properties of the tested steels at the different cooling rates: (**a**) tensile strength; (**b**) yield strength; (**c**) elongation after fracture.

After the tested steels were cooled at different rates and subjected to tensile tests, the mechanical properties were obtained, which are shown in Figure 9. Though the tensile strength and yield strength of the 4 test steels all show a decreasing trend with the increase of cooling rate (Figure 9a,b), the decreasing rates of S3 and S4 are significantly lower than that of S1 and S2. In particular, as the cooling rate increases from 0.0038 to 0.0094 °C/s, the yield strength of S3 and S4 decreases by only 3 and 9 MPa respectively, while the yield strength of S1 and S2 decreases very significantly.

Compared with S1 steel, the overall trend of tensile strength in the S3 steel without Mo or V and containing B is weaker, which may be related to the precipitation of Mo and V carbonitride, and the yield strength is obviously higher than that of S1 steel. Compared with S2 steel, the tensile strength and yield strength of S4 steel added with B are both higher. This is because B reduces the temperature and driving force of the austenite to ferrite transformation and promotes the generation of acicular ferrite and bainite (Figure 6), which thereby plays an important role in structure strengthening.

Thus, the above experimental results suggest the composition optimization can significantly improve the cooling sensitivity of this type of steel. In addition, compared with S1 and S2 steels, the elongation rates of S3 and S4 at different cooling rates are relatively stable (Figure 9c).

**Figure 10:**
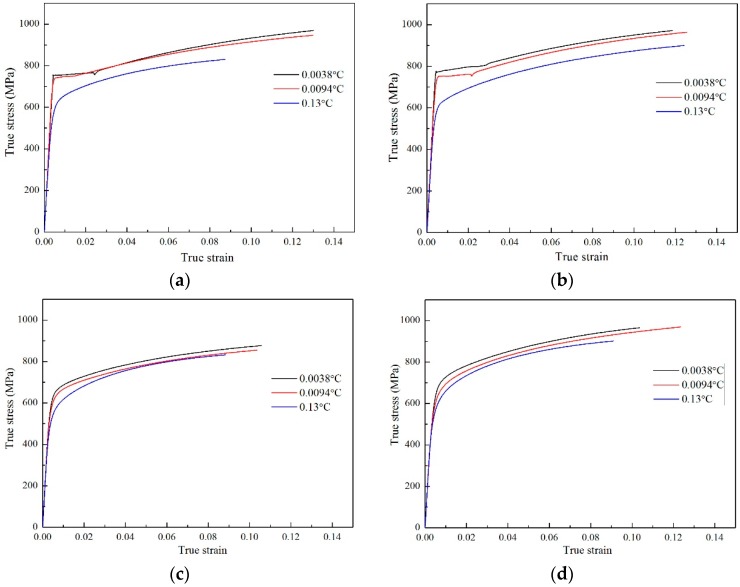
Tensile curves of the tested steels at the different cooling rates: (**a**) S1 steel; (**b**) S2 steel; (**c**) S3 steel; (**d**) S4 steel.

Figure 10 shows the true stress–strain curves of the tested steels at the cooling rate of 0.0038 °C/s, 0.0094 °C/s, and 0.13 °C/s, respectively. It can be seen from Figure 10 that, for each tensile specimen, the peak stress decreases with the increase in cooling rate. It may be caused by the decrease of the precipitation amount as the cooling rate increases. Furthermore, it can be found in Figure 10 that, for S3 and S4 steel, the true stress–strain curves are very close to each other, contrary to that of S1 and S2 steel. This phenomenon may be due to the addition of B in S3 and S4, which promotes the formation of the acicular ferrite and bainite and therefore improves the strength of the tested steels. Consequently, the strengths of S3 and S4 are less sensitive to the cooling rate than that of S1 and S2.

## 4. Discussion

### 4.1. Hot Crack Formation Mechanism of Continuous Casting Billets

The cracks in the third brittle zone are usually surface cracks, which is because the high temperature plastic deformation caused by the thermal stress during the cooling process of the slab exceeds the fracture limit of the material. The thermal strain can be calculated from Equation (1), where *ε_γ_* is the thermal strain in austenite, and *ε_α_* is the thermal strain in ferrite.

(1) $$\varepsilon =\varepsilon_{\gamma }+\varepsilon_{\alpha }$$

Since the strength of ferrite is far weaker than that of austenite at a certain temperature, the strain is concentrated in ferrite when austenite and ferrite coexist [25]. Thus, Equation (1) can be substituted for Equation (2), where *T_α_* is the initial temperature of ferrite transformation, and $\varepsilon_{\gamma }^{T_{\alpha }}$ is the thermal strain accumulated in austenite by the end of ferrite transformation initial temperature.

(2) $$\varepsilon =\{\begin{array}{cc} \varepsilon_{\gamma } & T>T_{\alpha }\\ \varepsilon_{\gamma }^{T_{\alpha }}+\varepsilon_{\alpha } & T<T_{\alpha } \end{array}$$

Noticeably, the above thermal strain values occur in the whole volume of each specimen and are thus called the nominal thermal strain. The thermal strain occurring in the ferrite phase is named as the real thermal strain and can be calculated by Equation (3), where *f* is the volume fraction of ferrite.

(3) $$\varepsilon^{’}{}_{\alpha }=\varepsilon_{\alpha }/f$$

Based on thermal expansion curves, the initial temperature and terminal temperature of austenite-to-ferrite transformation are determined (Table 2). At the end of the austenite to ferrite transformation, the volume fraction of ferrite is supposed to be 100%. Based on the Lever Rule and the fracture temperature, the ferrite volume fraction is calculated at break, shown in Table 5.

The thermal strain is simulated through the constant speed tensile under continuous cooling, and the cooling and transformation start from 910 °C at the same time with a cooling rate of 0.01 °C/s and a deformation rate of (6 mm/11,000)/s. Thus, when fracture occurs, the fracture strain and the strains accumulated in austenite and ferrite can be calculated by Equations (4)–(6), where *T_c_* is the fracture temperature, and *L* is the gauge length.

(4) $$\varepsilon^{c}=\frac{6}{11000}\times \frac{910-T_{c}}{0.01\times L}$$

(5) $$\varepsilon_{\gamma }^{T_{\alpha }}=\frac{6}{11000}\times \frac{910-T_{\alpha }}{0.01\times L}$$

(6) $$\varepsilon_{\alpha }^{c}=\frac{6}{11000}\times \frac{T_{\alpha }-T_{c}}{0.01\times L}$$

**Figure 11:**
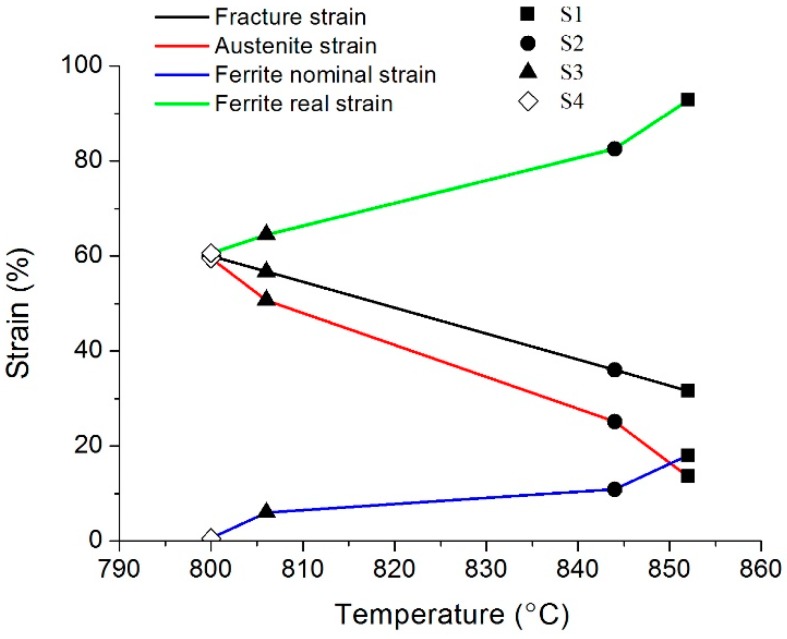
The $\varepsilon^{c}$ (fracture strain), $\varepsilon_{\gamma }^{T_{\alpha }}$ (austenitic strain), $\varepsilon_{\alpha }^{c}$ (ferrite nominal strain), and $\varepsilon_{\alpha }^{’c}$ (ferrite real strain) calculated by Equations (3)–(6), as well as their relations with the initial γ→α transformation temperature.

The results based on Equations (3)–(6) are listed in Figure 11, including the $\varepsilon^{c}$ (fracture strain), $\varepsilon_{\gamma }^{T_{\alpha }}$ (austenitic strain), $\varepsilon_{\alpha }^{c}$ (ferrite nominal strain), and $\varepsilon_{\alpha }^{’c}$ (ferrite real strain), as well as their relations with the initial transformation temperature of γ→α. Clearly, as the temperature of austenite to ferrite transformation decreases, the fracture strain gradually increases, indicating the drop of ferrite transformation temperature can inhibit the formation of hot cracks. Moreover, the ferrite real strain calculated by Equation (3) is far larger than the ferrite nominal strain calculated by Equation (6), as shown in Figure 11, suggesting a severe strain concentration in the ferrite [25]. When austenite and ferrite are coexistent, since the ferrite is far weaker than austenite, the strain concentration in ferrite may be one of the causes for the formation of cracks in the intergranular ferrite. This speculation is supported by the metallographic observations (Figure 2).

The experimental results are consistent with the conclusions of other studies [26,27], which are that if abundant ferrite (>45%) exists before deformation or numerous ferrite forms during deformation close to 883 °C, then good hot ductility can be obtained, which effectively avoids crack formation. This is because at a higher ferrite concentration, the ferrite real strain ($\varepsilon_{\alpha }^{’c}$) is smaller, leading to the smaller tendency of strain concentration and the lesser likelihood of cracking in the cast billets.

Moreover, the addition of B into S3 and S4 steels significantly lowers the austenite-to-ferrite transformation temperature and inhibits the formation of intergranular ferrite, so the crack formation due to strain concentration at the straightening process of continuous casting billets is avoided. Furthermore, the addition of B can make the ductility troughs tend to be flat or even disappear [16], and consequently, can considerably improve the hot ductility of continuous casting billets. As reported [12], the improvement of hot ductility in B-containing microalloyed steels is also related to the enhancing grain boundary cohesion, leading to an easier flow in the austenite lattice, where the grain boundary segregation and precipitation of B have an important role.

### 4.2. Mechanical Properties of Hot Rolled Coil

Figure 9b shows the effects of cooling rate on the yield strength. Clearly, as the cooling rate increases, the yield strength of all four types of steels decreases, which is characteristic of the precipitation strength steels [28]. This is because the quickened cooling rate allows carbonitride to stay at a suitable precipitation temperature for a short period of time and inhibits the formation of the precipitate phase, so the potential in the precipitation strengthening of carbonitrides is restrained [29]. This result is consistent with the finding that the amount of TiC precipitation is gradually decreased with the increasing of cooling rates observed in Figure 7, so the precipitated phase can be easily inhibited by the increment of cooling rates.

Moreover, the strengths of S3 and S4 steels are significantly less sensitivity to cooling rates than S1 and S2 (Figure 9 and Figure 10). This is because the addition of B into S3 and S4 indeed promotes the formation of bainite. When the cooling rate increases, the tendency to form acicular ferrite and bainite surpasses in S3 and S4 over S1 and S2 steels (Figure 5, Table 4), but the acicular ferrite and bainite are both stronger than the equiaxed ferrite, and this structure strengthening can compensate for the loss of precipitation strength to some extent, thereby reducing the cooling rate sensitivity of S3 and S4 steels.

Thus, the structure strengthening provided by acicular ferrite and bainite can be utilized, or appropriate ageing time to fully precipitate the microalloy carbonitride should be applied, so as to modify the relatively low strength at the head and end of hot rolled coils.

### 4.3. Rationale of Physical Metallurgy

The composition of four alloy elements (Ti, B, Mo, V) in micro-alloyed steels was optimized so as to improve the strength at the end and head of hot rolls, and the cracking of casting slabs.

During the whole technological process, four types of carbonitride may be precipitated from micro-alloyed steels, including Ti(CN), NbC, Mo_2_C, and VC. The formation order of these precipitates is determined by the affinity between metal atoms and the interstitial atoms C and N, in order of Ti(CN), NbC, Mo_2_C, and VC [20]. Among them, Ti(CN) particles are the most stable and usually precipitate on the solid/liquid interface during the metal solidification or in the hot austenite during the processing. Due to the large grain size, Ti(CN) contributes little to the precipitation strength, but can refine austenite grains well. The Ti(CN) has a strong stability, and once precipitated, it usually does not dissolve any more in the subsequent reheating process and fixes the interstitial atoms C and N. Therefore, usually in the steels added with Ti, the solid solution concentration of N is very low, and that of C also declines significantly.

The NbC, Mo_2_C, and VC may precipitate in ferrite, thereby contributing to the precipitation strengthening, but also can compete with each other. If the C concentration is not enough high, Mo_2_C or VC will not precipitate, so the precipitation strengthening may not be improved even after the addition of Mo and V. Moreover, the Mo, V, and Ti are all strong carbonitride formation elements and can raise the γ→α transformation temperature and lower the transformation driving force. Si as a typical α stabilizer can raise the γ→α transformation temperature [30]. Based on the above conclusions, compared with S1 steel, S2 steel contained no Mo or V and increased the Si content 0.1% to 0.8%, and the effects of these two changes on the γ→α transformation temperature were repelled by each other. Thus, it is reasonable that the beginning and ending temperatures of γ→α transformation and the fracturing temperature are slightly lower than those of S1 (Table 2 and Table 3). The phenomena in S3 and S4 can also be explained by the above reasons.

The Ti concentration declines from 0.11% to 0.08%, which releases a part of C atoms that are fixed previously and increases the C solid solution concentration, so as to lower the γ→α transformation temperature, which is favorable for improving the hot ductility of steels (Figure 11) and reducing the cracking sensitivity of the casting slabs. On the other hand, the rise of C solid solution concentration can also enhance the precipitation driving force of NbC in ferrite and lead to the left shift on the precipitation kinetics curve, which lowers the cooling rate sensitivity of the steels and is favorable for enhancing the strength at the head and end of hot rolled coils. Finally, the lower Ti concentration also decreases the amount of carbonitride precipitation during the solidification and the average sizes of Ti(CN) particles, which is advantageous to improve the high-temperature and room-temperature ductility and room-temperature and low-temperature impact ability of this type of steels.

Moreover, the steels were added with 0.002% B, which did not exist in the steels previously. B has a strong ability to inhibit the formation of intergranular ferrite and can significantly improve the stability of supercooled austenite, so as to effectively prevent the formation of surface transverse cracks [12,13]. The addition of B also promoted the formation of acicular ferrite and bainite, and thus this structure strengthening can be utilized to compensate for the loss of precipitation strengthening, which improved the strength at the head and end of hot rolled coils.

Also, the original Mo and V were eliminated. This is because at the current C solid solution concentration, Mo_2_C and VC are at a disadvantage in the competition with NbC precipitation reaction and thus cannot fully precipitate, so they cannot contribute to the precipitation strengthening. Moreover, since Ti, Mo, and V can delay the bainite transformation, the removal of Mo and V and the decreased Ti concentration are favorable for the formation of bainite. Though the solid solution state Mo promotes the formation of acicular ferrite, B is already added and is more effective than Mo in this aspect.

Generally, the composition optimization can solve the cracking of casting slab and low end-head strength of this type of steels without changing the existing production processes and can also reduce the alloy costs.

## 5. Conclusions

(1) At the deformation rate of (6 mm/11,000)/s, steels containing 0.002% B showed adequate hot ductility with reduction of area (RA) values > 60%, while that containing B free exhibited poor hot ductility with RA values < 40%.

(2) As the cooling rate increasing from 0.0094 to 0.13 °C/s, TiC precipitate was retained, and the amount of TiC gradually reduced, resulting in the lower tensile properties. At the same cooling rate, steels containing B showed much more bainite volume fraction than that containing B free, and higher tensile properties.

(3) The addition of B stabilizes the super-cooling austenite and lowers the austenite to ferrite transformation temperature, so as to inhibit the formation of intergranular ferrite and reduce the crack sensitivity of continuous casting billets.

(4) During hot deformation and cooling, strain concentration, applied on the low-strength α phase, is the mainly reason of surface transverse cracking of slab in the high temperature of α + γ two phase region.

(5) During the cooling of steels, the increasing of the cooling rate inhibits the precipitation of carbonitride and thereby weakens the effect of precipitation strengthening. Thus, the relatively low strength at the head and end of hot rolled coils is caused by the weaker precipitation strength, due to the fast cooling at the head and end during the coiling and cooling processes.

(6) The optimization of alloy composition can enhance the valid C solid solution concentration and promote the formation of strong acicular ferrite and bainite. Thus, this method can be used to compensate for the weak precipitation strengthening during the fast cooling, or an appropriate aging process can be adopted to reinforce the precipitate phase, so as to control the strength of this type of steel.

## Figures and Tables

**Table 1:** Chemical composition of experimental steels (in wt.%).

Steel	C	Si	Mn	Nb	Ti	Mo	B	Al	V	S
S1	0.071	0.10	1.847	0.047	0.112	0.163	-	0.0365	0.0164	0.0007
S2	0.071	0.78	1.807	0.047	0.117	-	-	0.0306	-	0.002
S3	0.071	0.11	1.936	0.046	0.085	-	0.0024	0.0324	-	0.003
S4	0.071	0.8	1.903	0.048	0.088	-	0.002	0.0387	-	0.002

**Table 2:** Temperatures of austenite to ferrite transformation.

Steel	Starting Temperature/°C	Ending Temperature/°C
S1	885	715
S2	864	713
S3	817	698
S4	801	680

**Table 3:** Results of the hot tensile testing.

Steel	Elongation/%	RA/%	Fracture Temperature/°C
S1	31.6	35.1	852
S2	36	40	844
S3	56.3	62.6	806
S4	>61.1	>65.43	<800

**Table 4:** Bainite volume fraction of experimental steels at different cooling rate.

Cooling Rate/(°C/s)	S1	S2	S3	S4
0.0038	1.9%	0.5%	5.3%	6.1%
0.0094	14.4%	10.3%	32.1%	44.8%
0.13	58.6%	67.2%	72.1%	89.8%

**Table 5:** The ferrite volume fraction of the tested steels at fracture.

Steel	S1	S2	S3	S4
Ferrite volume fraction/%	19.4	13.2	9.3	0.9
